# Supervised and unsupervised learning to classify scoliosis and healthy subjects based on non-invasive rasterstereography analysis

**DOI:** 10.1371/journal.pone.0261511

**Published:** 2021-12-23

**Authors:** Tommaso Colombo, Massimiliano Mangone, Francesco Agostini, Andrea Bernetti, Marco Paoloni, Valter Santilli, Laura Palagi

**Affiliations:** 1 Department of Computer, Control and Management Engineering Antonio Ruberti, Sapienza University of Rome, Rome, Italy; 2 Department of Anatomical and Histological Sciences, Legal Medicine and Orthopedics, Sapienza University of Rome, Rome, Italy; 3 aHead Research ETS, Rome, Italy; University of Illinois at Urbana-Champaign, UNITED STATES

## Abstract

The aim of our study was to classify scoliosis compared to to healthy patients using non-invasive surface acquisition via Video-raster-stereography, without prior knowledge of radiographic data. Data acquisitions were made using Rasterstereography; unsupervised learning was adopted for clustering and supervised learning was used for prediction model Support Vector Machine and Deep Network architectures were compared. A *M*-fold cross validation procedure was performed to evaluate the results. The accuracy and balanced accuracy of the best supervised model were close to 85%. Classification rates by class were measured using the confusion matrix, giving a low percentage of unclassified patients. Rasterstereography has turned out to be a good tool to distinguish subject with scoliosis from healthy patients limiting the exposure to unnecessary radiations.

## Introduction

Adolescent idiopathic scoliosis (AIS) is a three-dimensional deformity of the spine, which is characterized by deformation of vertebral column curvatures on the sagittal, frontal and transverse plane. X-ray are used to diagnose AIS, as they allow to detect vertebral rotation and to compute Cobb angle, needed for AIS classification. X-rays, however, carry health risk from repetitive exposure to ionizing radiation [1] and cannot aid the physician to detect postural changes associated to AIS.

The use of Magnetic resonance imaging (MRI) may be also recommended for adolescents experiencing atypical characteristics of idiopathic scoliosis because it can show abnormal tissue areas around the spine [2]. On the other hand the routine use of MRI significantly increases health-care costs significantly and may reveal mild variations from normal findings without clinical relevance, which can influence decision-making [3, 4].

Postural assessment with the study of the “static” standing posture, represents a relevant issue in the daily practice of physicians dealing with back diseases, especially those involving children and adolescents, in whom particular attention should be paid to the correct growth of their developing body. Nowadays physicians usually perform a postural evaluation based on a clinical examination, using their own experience and clinical examination Like Adams test that is used to detect deformities as well as asymmetries between the two sides of the body. One of the main difficulties is to identify postural parameters eligible for the final diagnosis of scoliosis and those that are between normal and pathological and cannot be applied as diagnostic criteria.

The need to have a non-invasive examination with three-dimensional characteristics has prompted researchers to use different methods to detect postural abnormalities analyzing the surface of the back [5] through different mathematical methods reconstructing digitally the spine.

The need to have a non-invasive examination with three-dimensional characteristics has prompted researchers to use different methods to detect postural abnormalities analyzing the surface of the back [5]through different mathematical methods reconstructing digitally the spine. Grünwald et al. [6] presented an approach to evaluate scoliosis from the three-dimensional image of a patient’s torso, captured by an ionizing radiation-free body scanner, in combination with a model of the ribcage and spine. Recently, Video-Raster-Stereography (VRS) has been proposed as an objective non-invasive method for instrumented three-dimensional (3D) back shape analysis and reconstruction of spinal curvatures and deformities without radiation exposure [7, 8].

The main drawback with the application of VRS to clinical practice like in AIS screening, is represented by the lack of a codified system to analyze and interpret the whole number of parameters derived from any single acquisition.

VRS is based on multiple stereophotogrammetric surface measuring of the back. For each acquisition of the surface topography, it processes more than one-hundred different quantitative parameters concerning the 3D subject’s posture, thus each parameter representing information on one of the three planes of space. However, in the clinical use of VRS applied to scoliosis, doctors refer mainly to a few information relating to the frontal plane. This is mainly due to the difficulties in dealing the large number of VRS parameters for which there are no certified reference values to be used to identify normal/abnormal situations, often resulting in a subjective interpretation of objective data. Indeed, the analysis of data from either a single patient or a population of subjects is one of the most critical issues in modern medicine. Despite technological advances, the large amount of data could be difficult to understand and could slow down the diagnostic and therapeutic approach, potentially causing unpleasant consequences for patients and operators. Nowadays, Data Mining (DM) and more specifically Machine Learning (ML) techniques have obtained much interest in the medical field to obtain relevant information from different medical data sets. Differently from classical statistical parametric inference, ML fits in the class of inductive statistical methods that infers from data both the model and its parameters and allows modeling nonlinear and multivariate relationships. In non-parametric models, no assumptions are set on the ground-truth, namely on the underlying distribution or on relationships among data. ML models can be particularly useful in the definition of complex multivariate mappings when there is no evident simple relationship among the large number of parameters. The use of these techniques in medical areas is changing the way to approach the patients, because they could simplify and accelerate the clinical processes [9, 10]. Indeed, they are increasingly being used to study problems related to the spine, mostly in radiological imaging [11, 12]. Recently, Chen et al. in 2021 [13] realized a narrative review describing the application of ML in clinical practice procedures regarding scoliosis, including screening, diagnosis, and classification, surgical decision making, intraoperative manipulation, complication prediction, prognosis prediction, and rehabilitation. They highlighted that an accurate diagnosis with ML can help surgeons avoid misjudgment.

Jaremko et al. [14] was the first to use neural networks to correlate spine and rib deformities in scoliosis. The investigators compared artificial neural networks (ANNs) and linear regression to predict rib rotation, with the results that ANNs averaged 60% correct predictions compared to 34% for linear regression analysis. In [15], a support vector machine (SVM) classifier has been used for combining surface topography of human backs and clinical data to assess the severity of idiopathic scoliosis.

Recently, there is a growing interest in developing reliable and noninvasive methods based on VRS to monitor the three-dimensionalm(3-D) progression of scoliosis [16]. Indeed, in the clinical use of VRS applied to scoliosis, doctors refer mainly to a few information relating to the frontal plane. It should be emphasized that the scoliotic deviation involves deviations on all planes of the space. Therefore the identification of “patterns” of data that considers conjointly the coronal, sagittal and axial deviations could be fundamental in clinical practice.

We believe that ML could constitute a fundamental analytical tool for accounting relationships on the three planes of space that are not taken into consideration in normal clinical routine. Indeed, ML models can be particularly useful in the definition of complex multivariate mappings such as in the case of the relationships between a large number of VRS features and postural diseases such as scoliosis.

In this paper, we want to develop a prediction tool to classify AIS subjects from healthy ones, using only rasterstereographic measurements as parameters. Particularly, we want to verify whether it is possible to select a limited number of these parameters which can be identified as those that bring most of the information and that can be used by physicians in clinical practice to distinguish between AIS subjects and healthy ones. With this aim, the objective of the present study has been to apply unsupervised and supervised ML techniques to gain insight into the information drawb by the rasterstereographic measurements.

## Materials and methods

We designed a retrospective study and the study protocol was approved by the Ethics and Experimental Research Committee of the Umberto I University Hospital—Sapienza University, Rome, Italy (Rif. 6221, Prot. 0104/2021). All procedures performed in studies involving human participants were in accordance with the ethical standards of the institutional and / or national research committee and with the Helsinki Declaration of 1964 and its subsequent amendments or comparable ethical standards. Informed consent was obtained from all individual participants included in the study. Participants’ informed consent was obtained in written form, using the format required by the ethics committee.

The research has been conducted by the Department of Physical Medicine and Rehabilitation (PMR) for the acquisition of data (the patient selection, the postural evaluation, the scoliosis/healthy diagnosis and the video-raster-stereography acquisition) in cooperation with the Department of Computer, Control, and Management Engineering (DIAG) “Antonio Ruberti” for the construction of learners and features extraction—both research groups are from Sapienza University of Rome. The overall process required continuous interaction between the two groups of researchers.

### Video-raster-stereography acquisition of data

The available data are constituted by theVideo-Raster-Stereography (VRS) measures of subjects who have undergone a clinical check and have been diagnosticated as healthy/AIS. Acquisition of data was performed through VRS by the Formetric^™^4D system (Diers International GmbH, Schlangenbad, Germany).

Briefly, the system consists of a light projector which projects a line grid on the back surface of the undressed patient which is recorded by an imaging unit. The three-dimensional back shape leads to a deformation of the parallel light lines, which can be detected by a camera positioned at a different angle from the projector (triangulation system). Using a standardized mathematical analysis, the following specific landmarks are automatically determined by assigning concave and convex areas to the curved light pattern:

(i) the spinous process of 7th cervical vertebra (Vertebra Prominens—VP);
(ii) the spinous process of 12th thoracic vertebra (Th12);
(iii) the midpoint between the lumbar dimples;
(iv) the cervical-thoracic inflexion point (ICT);
(v) the thoracic-lumbar inflexion point (ITL);
(vi) and the lumbar-sacral inflection point (ILS).

The patient is asked to stand still in an upright posture at a fixed distance from the camera for 6 seconds, during which a total number of 12 scans are performed. The mean value of the 12 measures is reported as output.

**Fig 1:**
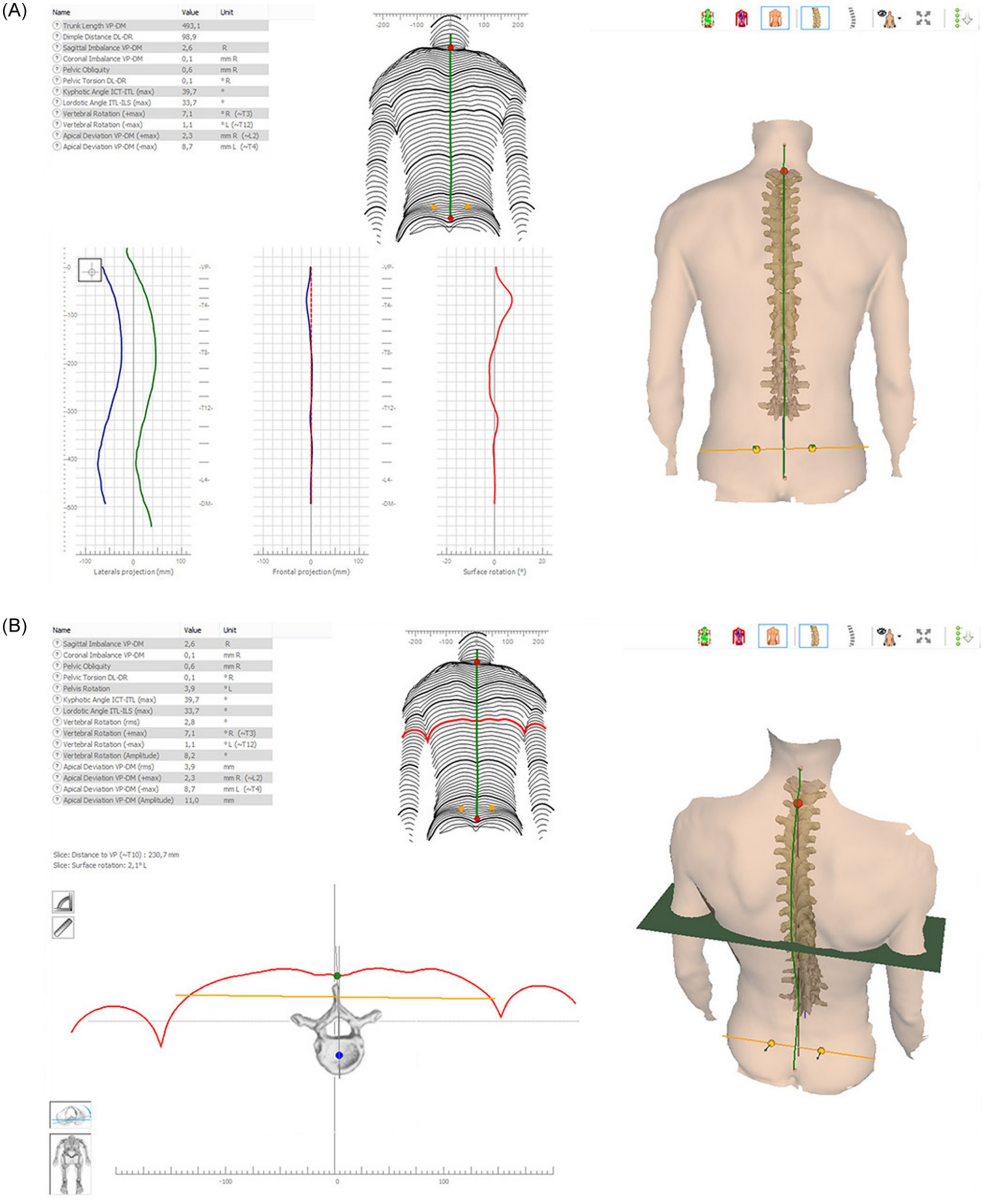
Formetric’s output representation (from https://diers.eu).

Based on these landmarks, a three-dimensional model of the whole surface, comparable to a plaster cast, the sagittal profile, and shape parameters describing this profile are generated. Example of pictures accompanied by table of measures obtained by Formetric^™^4D are reported in the Fig 1.

The accuracy of such measures and Formetric^™^ functioning can be found in [17, 18]. Derived parameters from automatic landmarks are, among the others, thoracic kyphosis angle, lumbar lordosis angle, lumbar fléche, cervical fléche and kyphotic apex as described by Stagnara [19, 20].

For each patient, the total number of VRS features calculated by Formetric^™^is 40. The features are all numerical and the full list is reported in Table 1 together with the units of measure.

**Table 1:** The full list of Formetric^™^ features.

Feature	Unit of Measure	Feature	Unit of Measure
Trunk length_VP-DM	mm	Lumbar Fléche_(Stagnara)	mm
Trunk length_VP-SP	mm	Kyphosis angle_ICT-ITL	degree
Trunk length_VP-SP	%	Kyphosis angle_VP-ITL	degree
Dimple distance-DR	mm	Kyphosis angle_VP-T12	degree
Dimple distance_DL-DR	%	Lordotic angle_ITL-ILS_(max)	degree
Trunk inclination_VP-DM	degree	Lordotic angle_ITL-DM	degree
Trunk inclination_VP-DM	mm	Lordotic angle_T12-DM	degree
Lateral_flexion_VP-DM	degree	Pelvic inclination	degree
Lateral_flexion_VP-DM	mm	Surface rotation_(rms)	degree
Pelvic obliquity_DL-DR	degree	Surface rotation_(max)	degree
Pelvic obliquity_DL-DR	mm	Surface rotation_(+max)	degree
Pelvic torsion_DL-DR	degree	Surface rotation_(-max)	degree
Pelvic inclination_(dimple)	degree	Surface rotation_(width)	degree
Pelvis rotation	degree	Pelvic torsion	degree
Inflexion point_ICT	mm	Lateral deviation_VPDM_(rms)	mm
Kypothic apex_KA_(VPDM)	mm	Lateral deviation_VPDM_(max)	mm
Inflexion point_ITL	mm	Lateral deviation_VPDM_(+max)	mm
Lordotic apex_LA_(VPDM)	mm	Lateral deviation_VPDM_(-max)	mm
Inflexion point_ILS	mm	Lateral deviation_(width)	mm
Cervical Fléche_(Stagnara)	mm	Pain_index_(Dr_Weiss)_rel	number

### Data description and preprocessing

Rasterstereographic collection of data was conducted for clinical purposes in the Department of PMR of Sapienza during the period January 1^*st*^, 2010—December 31^*st*^, 2016. Subject have been selected according to the following inclusion criteria: (i) male or female and (ii) age between 14 and 30. We excluded subjects with: (i) clinical history of congenital/acquired pathological condition of vertebrae (e.g. Scheuermann’s disease, spondylolysis, spondylolisthesis); (ii) history of vertebral fractures and/or vertebral surgery; (iii) diagnosis of disc protrusion/hernia at any spinal level; (iv) diagnosis of scoliosis secondary to neurological, rheumatological and/or congenital conditions; (v) diagnosis of AIS with Cobb angle measured on X-rays > 45 degrees; (vi) diagnosis of any neurological and/or rheumatological conditions.

Once analysed inclusion and exclusion criteria of patients screened for eligibility, a total of 298 subjects has been enrolled. In particular patients enrolled with diagnosis of scoliosis were 272 (∼ 90% of total) and healthy subjects were 26. The number of healthy/scoliotic subjects is strongly imbalanced and this is a well-known cause of bias in the learning process [21].

The data set is constituted by the VRS records of measures of the enrolled subjects. Each VRS record together with the corresponding status (health/AIS) of the subject defines a sample of the data set. No other clinical or personal information are collected. For each patient Formetric^™^ returns more than one record, each representing a sample in the dataset. In particular patients enrolled with diagnosis of scoliosis had a total of 1111 Formetric^™^ records and Healthy patients had a total of 194 Formetric^™^ records.

Standard procedure to overcome drawbacks due to imbalance data requires under/over sampling. However, in the case of the Formetric^™^, we can exploit the nature of the data itself. Indeed, different records refer to the same patients and are obtained in a single measurement of the Formetric^™^. Hence, we decide to reduce imbalance by reducing the samples referring to scoliotic patient by simply averaging the different Formetric^™^ records that we had for each of the scoliotic patient (that were about 4 for each subject). At the end we obtained 272 AIS averaged samples and 194 Formetric^™^ healthy samples.

Finally the target set is obtained by merging samples of the two population of AIS patients (averaged) and healthy subjects (not averaged), so that we obtain a dataset made up of *m* = 466 samples each represented by the 40 Formetric^™^ features and a label in {−1, 1} that corresponds to healthy/scoliotic status. We summarize the main statistics of the target set in Table 2.

**Table 2:** Summary of descriptive statistics on the dataset.

Acquisition date	2010—2016
Number of distinct patients	298
Healthy Male/Female	17/9
Scoliosis Male/Female	118/154
Healthy/scoliosis ratio of patients	0.1
Number of samples after balancing	466
Number of healthy samples after balancing	194
Number of AIS samples after balancing	272
Healthy/scoliosis ratio in the target set	0.7

As mentioned in the introduction before the learning phase, data must undergo a cleaning and feature selection phase which usually reduces both the number of samples and the number of features which can be redundant with respect to the learning aim. Indeed learning machines performance are influenced both by the number of samples and the number of features of the target set used for training (see e.g. the surveys [22, 23]).

Before undergoing a features selection phase done by a ML procedure, described in the *Features selection procedure* Section, data were briefly analysed for cleaning and scaling purpose. All data or references that could in some way allow the identification of the patient or of different categories (for example name, age, etc.) have been eliminated to obtain a totally anonymous sample (anonymization). Actually, the physicians recognized that some of the features obtained by the Formetric^™^ contained duplicate information, in the sense that they correspond to measures of the same quantity, expressed in different units (e.g. mm or degrees). Hence we decided to eliminate the duplicate measures and we finally got a number of distinct features equal to 33. The eliminated features are reported in Table 3.

**Table 3:** Duplicated features eliminated with physicians’ support.

Feature	Unit of Measure	Eliminated
Trunk inclination_VP-DM	degree	Y
Trunk inclination_VP-DM	mm	N
Lateral_flexion_VP-DM	degree	Y
Lateral_flexion_VP-DM	mm	N
Pelvic obliquity_DL-DR	degree	Y
Pelvic obliquity_DL-DR	mm	N
Kyphosis angle_ICT-ITL_(max)	degree	N
Kyphosis angle_VP-ITL	degree	Y
Kyphosis angle_VP-T12	degree	Y
Lordotic angle_ITL-ILS_(max)	degree	N
Lordotic angle_ITL-DM	degree	Y

‘Y’ = eliminated, ‘N’ = maintained

After this basic features’ reduction, a first run of classification using the tools described in the section of *Materials and methods* was performed. Results obtained by unsupervised and supervised classification presented inconsistencies. Indeed analyzing the obtained results, we understood that there were features related to trunk length, which were in turn tied to the age of the patient, that played a dominant role in classification and thus resulting in a wrong classification. Therefore, we decided to remove additional features that are strongly related to trunk length, reported in Table 4. Thus the number of total features in the target set was further reduced further to 27.

**Table 4:** Eliminated features since highly dependent on trunk length.

Feature	Unit of Measure
Trunk length_VP-DM	mm
Trunk length_VP-SP	mm
Trunk length_VP-SP	%
Dimple distance_DL-DR	mm
Dimple distance_DL-DR	%

Among the 27 remaining features, there were some still depending on trunk length that cannot be eliminated because they may bring useful information. For these features, reported in Table 5, we divided each value by the trunk length, obtaining an adimensional value. In this way the target set was not biased by the age of the patients.

**Table 5:** Features dependent on trunk length normalized by trunk length_VP-DM in mm.

Feature	Unit of Measure
Inflexion point_ICT	mm
Kypothic apex_KA_(VPDM)	mm
Inflexion point_ITL	mm
Lordotic apex_LA_(VPDM)	mm
Inflexion point_ILS	mm

At the end of this process we got a target set made up of *m* = 466 samples (referring to 299 patients) each characterized by *n* = 27 input features $x^{i}\in R^{27}$ which are reported in Table 6 with a statistical report and one output label in *y*^*i*^ ∈ {−1, 1} which identifies healthy/scoliotic samples. We denote the target set as

$$T=\{\left(x^{i},y^{i}\right)\in R^{n}\times \left\{-1,1\right\},i=1\cdots ,m\}$$

**Table 6:** List of features $x\in R^{27}$ of the clean data set.

Feature	Name	Unit	Mean ± standard deviation	[min, max]
*x*_1_	Trunk inclination_VP-DM	mm	11,61 ± 24,70	[-72,81, 124,17]
*x*_2_	Lateral_flexion_VP-DM	mm	-3,20 ± 10,26	[-33,61, 30,00]
*x*_3_	Pelvic obliquity_DL-DR	mm	0,64 ± 5,52	[-22,13, 43,14]
*x*_4_	Pelvic torsion_DL-DR	degree	0,47 ± 2,74	[-7,87, 11,13]
*x*_5_	Pelvic inclination_(dimple)	degree	20,86 ± 6,77	[2,11, 37,36]
*x*_6_	Pelvis rotation	degree	0,39 ± 3,47	[-13,56, 12,10]
*x*_7_	Inflexion point_ICT/trunk length_VP-DM	adim	0,00 ± 0,02	[-0,05, 0,04]
*x*_8_	Kypothic apex_KA_(VPDM)/trunk length_VP-DM	adim	-0,31 ± 0,06	[-0,46, 0,00]
*x*_9_	Inflexion point_ITL/trunk length_VP-DM	adim	-0,57 ± 0,07	[-0,72, 0,00]
*x*_10_	Lordotic apex_LA_(VPDM)/trunk length_VP-DM	adim	-0,74 ± 0,06	[-0,87, 0,00]
*x*_11_	Inflexion point_ILS/trunk length_VP-DM	adim	-0,89 ± 0,05	[-0,99, 0,00]
*x*_12_	Cervical Fléche_(Stagnara)	mm	55,73 ± 21,64	[0, 00, 133, 77]
*x*_13_	Lumbar Fléche_(Stagnara)	mm	41,74 ± 17,51	[-10,75, 96,77]
*x*_14_	Kyphosis angle_ICT-ITL	degree	48,55 ± 10,38	[0, 00, 72, 27]
*x*_15_	Lordotic angle_ITL-ILS_(max)	degree	41,19 ± 9,87	[20, 09, 67, 63]
*x*_16_	Pelvic inclination	degree	21,14 ± 8,82	[-3,92, 41,01]
*x*_17_	Surface rotation_(rms)	degree	4,14 ± 1,95	[0, 89, 13, 22]
*x*_18_	Surface rotation_(max)	degree	0,40 ± 8,28	[-20,88, 32,35]
*x*_19_	Surface rotation_(+max)	degree	5,22 ± 4,53	[-1,62, 32,35]
*x*_20_	Surface rotation_(-max)	degree	-5,47 ± 3,40	[-20,88, 2,46]
*x*_21_	Surface rotation_(width)	degree	10,69 ± 4,78	[2, 63, 35, 20]
*x*_22_	Pelvic torsion	degree	1,70 ± 5,35	[-31,87, 23,74]
*x*_23_	Lateral deviation_VPDM_(rms)	mm	5,18 ± 3,30	[0, 00, 22, 84]
*x*_24_	Lateral deviation_VPDM_(max)	mm	3,10 ± 9,90	[-26,08, 38,20]
*x*_25_	Lateral deviation_VPDM_(+max)	mm	7,28 ± 5,80	[0, 00, 38, 20]
*x*_26_	Lateral deviation_VPDM_(-max)	mm	-4,61 ± 4,56	[-26,08, 0,00]
*x*_27_	Lateral deviation_(width)	mm	11,72 ± 6,65	[0, 00, 46, 49]

### Statistical analysis

Before entering a feature selection phase using standard tools in machine learning, we performed basic statistical analysis of the target data. We report in Table 6, for each of the features, besides the name and unit, the mean value, the standard deviation and the range (minimum and maximum value). Further, to check if it is possible to identify a high degree of correlation either among pairs of input features or among input features and the output class, we perform a Pearson test on the target set T.

None of the features present a strong correlation with the output being the max score 0.59. However, some pairs of features are highly correlated with each other, i.e. they present a Pearson score greater than 0.8 (e.g *x*_5_ and *x*_16_ which represent different procedure of measuring the pelvic inclination). The full Pearson matrix is reported in Table 7 where variables with Pearson score greater than 0.8 are in boldface type in a green box. We use the identified relationships in connection with the features selection procedure in the next section. In Table 8 we report a summary of max/min indexes in the Pearson matrix.

**Table 7:** Pearson Correlation matrix among features: In a green box the most correlated ones.

	*x*_2_	*x*_3_	*x*_4_	*x*_5_	*x*_6_	*x*_7_	*x*_8_	*x*_9_	*x*_10_	*x*_11_	*x*_12_	*x*_13_	*x*_14_	*x*_15_	*x*_16_	*x*_17_	*x*_18_	*x*_19_	*x*_20_	*x*_21_	*x*_22_	*x*_23_	*x*_24_	*x*_25_	*x*_26_	*x*_27_	y
*x*_1_	-0.10	0.10	-0.02	0.18	-0.03	-0.26	-0.41	-0.06	0.06	-0.03	0.33	**-0.88**	-0.36	-0.22	0.18	0.10	-0.11	-0.04	-0.12	0.05	-0.17	0.19	0.08	0.12	-0.06	0.17	0.26
*x*_2_		-0.20	-0.33	-0.01	0.13	-0.04	0.10	0.09	0.06	0.09	-0.06	0.09	-0.05	-0.02	-0.02	-0.04	0.21	0.18	0.14	0.06	0.36	-0.02	-0.09	-0.08	-0.05	-0.03	0.05
*x*_3_			-0.14	0.05	-0.04	-0.06	0.05	0.04	0.05	0.04	-0.04	-0.09	-0.07	0.02	0.05	-0.01	0.01	0.06	-0.03	0.07	0.13	0.09	-0.00	0.03	-0.11	0.10	-0.04
*x*_4_				0.02	-0.16	0.02	0.00	0.01	-0.01	-0.04	-0.06	0.00	0.00	0.05	0.03	0.18	-0.14	-0.06	-0.16	0.07	-0.13	0.09	0.09	0.10	-0.02	0.10	0.11
*x*_5_					-0.09	-0.09	-0.20	-0.31	-0.38	-0.44	-0.50	-0.32	-0.26	0.72	**0.85**	0.42	-0.07	0.14	-0.27	0.34	-0.04	0.24	0.14	0.18	-0.00	0.14	0.59
*x*_6_						0.06	0.08	0.11	0.11	0.12	0.03	0.03	0.00	-0.11	-0.10	-0.07	0.27	0.26	0.18	0.10	0.24	-0.01	0.05	0.01	0.03	-0.01	-0.11
*x*_7_							0.31	0.19	0.18	0.15	0.07	0.02	0.11	-0.02	-0.03	0.04	0.24	0.25	0.09	0.16	0.15	0.08	-0.02	0.08	-0.14	0.15	-0.21
*x*_8_								0.74	0.73	0.70	-0.21	0.35	-0.09	0.03	-0.10	-0.12	0.13	0.06	0.10	-0.03	0.10	-0.04	0.09	0.03	0.11	-0.07	-0.38
*x*_9_									**0.90**	**0.86**	0.07	0.09	-0.15	-0.17	-0.20	-0.23	0.11	-0.03	0.17	-0.15	-0.01	-0.11	0.04	-0.05	0.12	-0.14	-0.39
*x*_10_										**0.96**	0.14	-0.01	-0.20	-0.27	-0.23	-0.25	0.11	-0.04	0.19	-0.18	-0.03	-0.09	0.07	-0.02	0.13	-0.11	-0.45
*x*_11_											0.19	0.11	-0.14	-0.29	-0.30	-0.34	0.14	-0.05	0.26	-0.25	-0.03	-0.17	0.03	-0.10	0.16	-0.20	-0.47
*x*_12_												-0.25	0.14	-0.65	-0.51	-0.35	-0.06	-0.23	0.16	-0.34	-0.10	-0.21	-0.08	-0.16	0.03	-0.13	-0.19
*x*_13_													0.44	0.12	-0.31	-0.32	0.05	-0.13	0.23	-0.30	0.13	-0.34	-0.12	-0.24	0.15	-0.32	-0.39
*x*_14_														0.09	-0.21	-0.30	0.00	-0.15	0.20	-0.29	-0.05	-0.40	-0.17	-0.34	0.13	-0.38	-0.14
*x*_15_															0.79	0.23	-0.02	0.09	-0.11	0.16	0.01	-0.01	0.01	-0.04	0.07	-0.11	0.37
*x*_16_																0.33	-0.04	0.13	-0.18	0.26	-0.00	0.16	0.12	0.11	0.05	0.05	0.48
*x*_17_																	-0.13	0.32	-0.65	0.79	-0.03	0.59	0.27	0.52	-0.17	0.54	0.46
*x*_18_																		0.83	0.70	0.23	0.48	-0.09	-0.25	-0.20	-0.20	-0.06	-0.08
*x*_19_																			0.31	0.69	0.49	0.24	-0.07	0.12	-0.29	0.27	0.18
*x*_20_																				-0.48	0.32	-0.49	-0.39	-0.55	-0.01	-0.47	-0.31
*x*_21_																					0.21	0.60	0.23	0.53	-0.26	0.61	0.41
*x*_22_																						0.07	-0.07	0.02	-0.16	0.15	-0.06
*x*_23_																							0.30	0.72	-0.34	**0.84**	0.26
*x*_24_																								**0.80**	0.67	0.22	0.08
*x*_25_																									0.21	0.69	0.16
*x*_26_																										-0.52	-0.10
*x*_27_																											0.20

**Table 8:** Summary of Pearson coefficients (absolute values).

	features vs features	features vs output
Max abs correlation	0.96	0.59
Min abs correlation	0.00	0.04
Avg abs correlation	0.19	0.26

### Classification models

In this section we briefly describe the three main classes of Machine Learning (ML) methods for classification used to analyze data in the target set. In particular, as we mentioned in the introduction, we are interested in using both unsupervised clustering and supervised classification. Unsupervised learning consists in detecting if samples can be split into groups, i.e. the clusters, which possess some similarities in a defined metric. We want to apply a clustering strategy as a first step to check how good is the information tied to the only features without driving the classification by the known status of the subject. In a second phase, we adopt a supervised learning procedure and we use as label the status (healthy or scoliotic) of the subject to derive the predictive model.

#### Unsupervised classification

Unsupervised learning does not use any a priori information on the labels of the target data, with the aim of grouping ‘similar’ samples (clusters) based on the features only. Similarity is usually measured by a metric distance between samples both intra-group and extra-group with the aim of maximizing the distance between samples in different clusters, while minimizing the distance between samples belonging to the same cluster. The known label *y*^*i*^ have been used in the ‘a posteriori’ analysis to evaluate the performance as explained in the next section. From a clinical point of view our aim in unsupervised clustering is to check whether the features selected contain enough ‘good’ information to allow a ‘natural’ split into two clusters. We select as clustering method an improved version of the basic K-Means [24, 25] algorithm called K-Means++ [26], where we set the number of clusters to *K* = 2 (healthy vs scoliosis). As metric distance *d*(*x*^1^, *x*^2^) between *x*^1^ and $x^{2}\;\in \;R^{n}$ we use the standard Euclidean norm $\sqrt{\sum_{i=1}^{n}{\left(x_{i}^{1}-x_{i}^{2}\right)}^{2}}.$

Results obtained by unsupervised learning compared to those obtained by supervised learning, can help in checking the existence of biases in the features.

Actually, biases appeared in the first stage of our study when samples were clustered on the height of the patient, being AIS patients mostly adolescents, thus inducing wrong clusters. We used information derived by this first clustering results to clean up the data. in particular, this led us to remove the features in Table 4 that are strongly related to trunk length and to normalize features in Table 5 depending on the on trunk length to avoid implicit misleading classification.

It is worth to mention that we performed the clustering procedure as a first step at the very beginning of the project and that results pointed out some bias in the data that induced wrong clusters. We used information derived by the first clustering results to clean up the data.

The results, reported in the corresponding section, highlight that the data can be sufficiently well clustered.

#### Supervised classification

In supervised classification the task is to learn from ‘labelled’ examples (*x*^*i*^, *y*^*i*^) *i* = 1, …, *m* as given in the target set T. For supervised learning, we consider two of the most famous models namely Support Vector Machines (SVM) [27–29] and Deep Networks (DN) [30]. SVM has been already used for detecting postural diseases tied to scoliosis in [9] with a different setting of data. In the postural field, DNs have been mainly used in image recognition to identify damaged vertebrae in the spine (see e.g. [31, 32]). Besides obtaining a good classifier to predict the class of new subjects, a side product of our research is the performance comparison in classification between SVM and DNs.

#### Support vector machines

Support Vector Machines (SVMs) are supervised binary classifiers in the class of kernel methods that learn the possibly nonlinear border between data belonging to different classes. We focus on nonlinear SVMs which define a possibly nonlinear decision function to predict the class of an unseen patients described by the features vector *x* as

$$class\left(x\right)=sign(\sum_{i=1}^{m}\alpha_{i}k\left(x^{i},x\right)+\beta ),$$

where *sign* is the sign function that returns value 1 or -1 (or zero) depending on the sign of the argument; *k*(⋅, ⋅) is a kernel function which represents a measure of similarity, i.e. a scalar product among data points in a transformed nonlinear space and $\alpha_{i}\in R$, *i* = 1, …, *m* and β∈R are the values to be set in the training phase.

Both linear and Gaussian kernels, see Table 9, were tested in this paper.

**Table 9:** Kernels used in the SVM experiments.

	Linear	Gaussian
*k*(*x*^*i*^, *x*) =	*x*^*T*^ *x*^*i*^	$e^{-\gamma \|x^{i}{-x\|}^{2}}$

The Gaussian kernel presents the hyper-parameter *γ* called the width of the kernel. Tuning of the hyper-parameters *γ* and *C*, which controls the ‘quote’ of misclassified training samples, have been done by means of grid search within a *M*-fold cross validation procedure.

#### Deep networks

**Fig 2:**
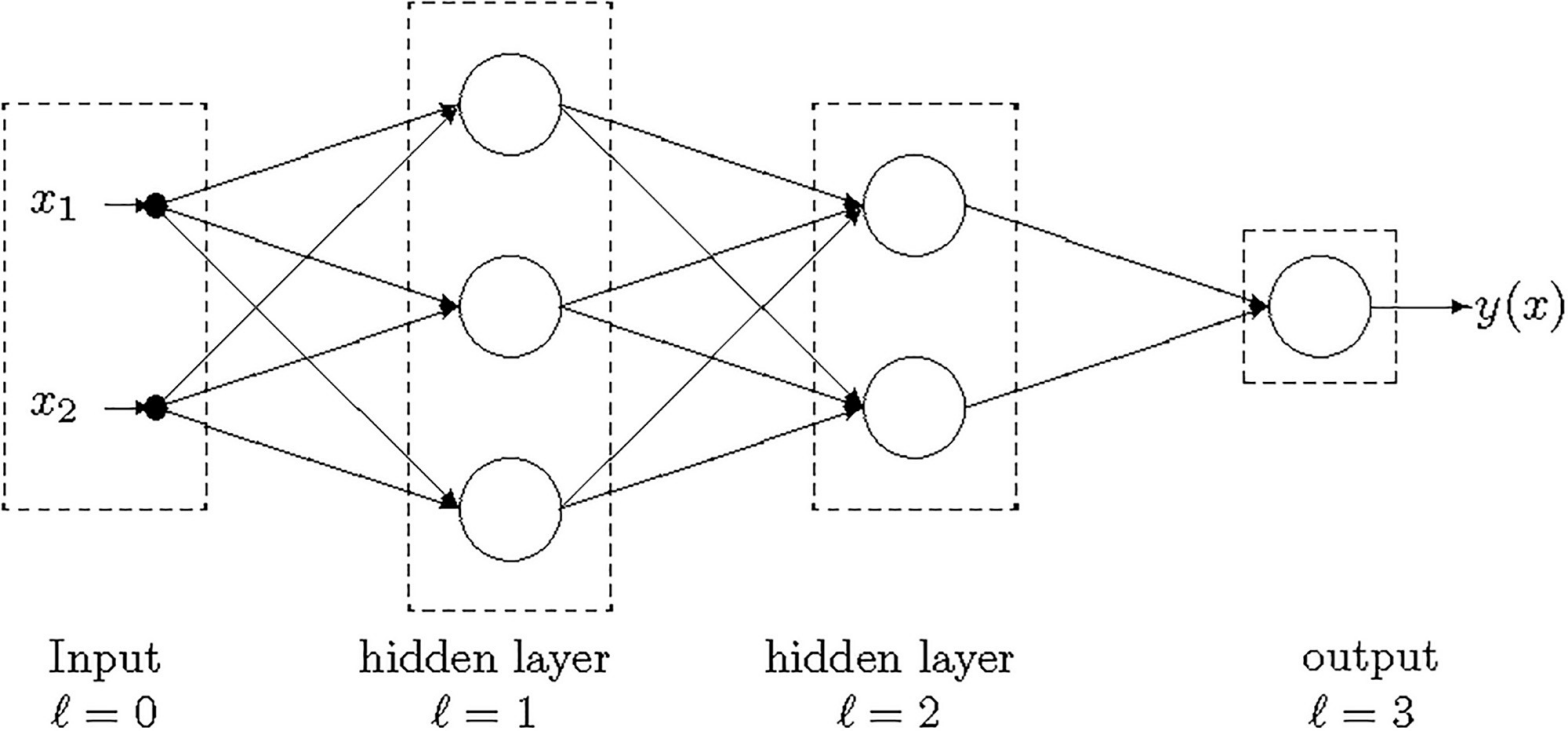
DN with two inputs (*n* = 2), two hidden layers (*L* = 3) and a single output.

The DNs [30] are multilayer feed-forward neural networks in which units (neurons) are organized into layers with forward connections from the input layer (*ℓ* = 0) to the output layer (*ℓ* = *L*) as reported in Fig 2.

Each unit in the hidden layers apply an activation function *g*(⋅) (that we assume be the same for all the neurons) to a weighted combination of the outputs of the units in the preceding layer. We use as activation function *g*(*z*) in the hidden units both the ReLU (Rectified Linear Unit) and the sigmoid reported in Table 10 as implemented in the ScikitLearn library [33] that we used in the tests. In the output layer we consider a sigmoid activation function tanh(⋅) that returns a value in {−1, 1}.

**Table 10:** Activation functions used in the DN experiments.

	ReLu	Sigmoid
*g*(*z*) =	max{0, *z*}	$\frac{e^{z}}{e^{z}+1}$

The hyper-parameters, number of layers *L* and neurons per layer *N*^*ℓ*^, *ℓ* = 1, …*L*, have been selected by a grid search within a *M*− fold cross validation procedure. The weights *W*^*i*^, *i* = 1, …, *L* are obtained by minimizing the *L*_2_ regularized mean square error.

### Performance measures

The ultimate task of a machine learning classifier is to give good performance on new unseen samples. This task is called generalization ability of a learner and it is in contrast with the perfect learning of the training data that leads to the so-called over-fitting phenomenon (see e.g. [23] and references therein). In order to check the performance of a learner without being biased by the learning process itself, usually the training phase is repeated by inserting some randomness in the process. In particular in the case of unsupervised learning, we use the full target set as training set and we repeat *M* times the K-means++ procedure [26], which has a random seed to start with, and we averaged the results.

To check the correctness of the obtained clusters $C_{i}$, *i* = 1, 2 at the end of each of the *M* runs of the training phase we use the known labels *y*^*i*^. In particular we measure the correctness of the clusters with the *purity* which is a simple and transparent accuracy measure. Each cluster is assigned to the class which is most frequent in the cluster, and accuracy is measured by counting the average number of correctly assigned samples. Formally we can write it as:

$$ACC_{purity}=\frac{1}{2m}\text{max}\{\sum_{i=1}^{m}|y^{i}+{\text{class}}_{i}|,\sum_{i=1}^{m}\left|y^{i}-{\text{class}}_{i}\right|\},$$

with

$${\text{class}}_{i}=\{\begin{array}{cc} +1, & x^{i}\;\in \;C_{1},\\ -1, & x^{i}\;\in \;C_{2}. \end{array}$$

In supervised learning, the target set is usually split into two parts: a training set, used only in the learning phase to train the machine, and a test set, used only in a post-learning analysis to quantify the generalization performance. In this way performance indicators of the learning machine are computed on subjects never shown to the learning process. However, when there are few available samples in the data set, as it happened in our case, it can be worthwhile to use a *M*-fold cross validation procedure which split randomly the target set into *M* subsets and train the learning machine on *M* − 1 subsets, leaving out one of them (called validation set) to compute the Key Performance Indicators (KPIs). The average of these KPIs over the *M* runs represents an estimation of the generalization performance.

The Key Performance Indicators (KPIs) to measure the quality of a classification machine used in this paper are:

- a 2×2 confusion matrix, where each elements represents the (averaged) percentage of classification of instances True Negative (%TN), True Positive (%TP), False positive (%FP) and False Negative (%FN) as shown below

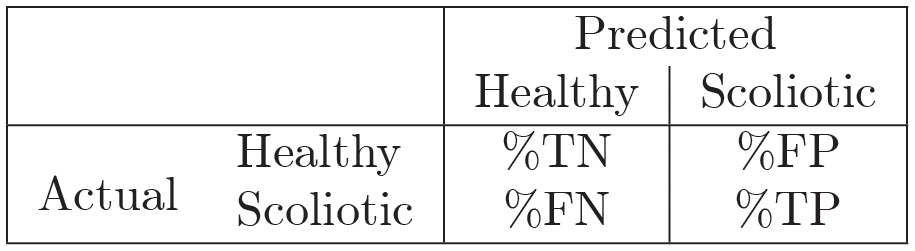
- classification accuracy (i.e. percentage of correct classified patients)
  $$\text{ACC}=\frac{TP+TN}{TP+FN+TN+FP}$$
  where TP, FN, TN, FP are the averaged number of instances classified as True Positive, False Negative, True Negative, False positive, respectively.
- Balanced Accuracy (BACC) attempts to account for the imbalance in classes and is the arithmetic average of the measure of accuracy on the solely positive cases (sensitivity/recall) and the accuracy on the solely negative cases (specificity)
  $$\text{BACC}=\frac{1}{2}[\frac{TP}{TP+FN}+\frac{TN}{TN+FP}]$$

The Key Performance Indicators (KPIs) are reported as an average over the *M* runs of the values obtained on the test set when supervised learning is used. The perfect learning corresponds to a 100% accuracy and it consists in having the sum over the diagonal of the confusion matrix being 100. This situation, whenever it appears, is in general untrustworthy being a signal of overfitting.

The training procedure both in the case of unsupervised and supervised learning can be summarized in the Algorithm 1.

**Algorithm 1 Training procedure and average evaluation of KPI**

1: Given the target set T given by *m*–by–(*n* + 1) = 466 × 28 samples;

2: **for** *k* = 0, 1, …, *M* **do**

3:  Extract randomly the training $T^{k}$ and the validation $V^{k}$ sets:

4:   for supervised learning $|T^{k}|=70\%\cdot m$ and $|V^{k}|=30\%\cdot m$;

5:   for unsupervised learning $|T^{k}|=100\%\cdot m$ and 0%;

6:  Train a classifier *f*^*k*^ using the training set $T^{k}$

7:  Compute the KPIs of the classifier

8: **end for**

9: Average the KPIs over the *M* runs

### Features selection procedure

A critical aspect for the success of any learning procedure stays in the reduction of the number of input features. It may happen that some features are redundant and/or add noisy information so that eliminating them will help the learning task. Further the features selection can give insight on which are the features that hold the most significant information and hence can give doctors indications about the key measures of Formetric 4D. To this aim we perform a feature selection phase before entering the true learning phase. In the literature, different methods for features selection have been proposed. We choose four different algorithms, described below, and we define a ranking of the features by assigning a vote based on how many algorithms have selected it.

Moreover we use this ranking to reduce training set dimension in the experiments to test whether the most selected features actually include the most significant patterns.

As tools for features selection, we used four different algorithms, both supervised and unsupervised, listed below:

1. L2-regularized SVM [28]
  $$\underset{w\in R^{n}}{\text{min}}{\left\|w\right\|}_{2}^{2}+C\sum_{i=1}^{m}\text{max}\left\{0,1-y_{i}\left(w^{T}x^{i}+b\right)\right\}$$
2. L1-regularized SVM [34]
  $$\underset{w\in R^{n}}{\text{min}}{\left\|w\right\|}_{1}+C\sum_{i=1}^{m}\text{max}\left\{0,1-y_{i}\left(w^{T}x^{i}+b\right)\right\}$$
3. Mutual information (MI) which is a non-negative value that measures the dependency between two random variables. The function relies on nonparametric methods based on entropy estimation from k-nearest neighbors distances as described e.g. in [35, 36].
4. Analysis Of Variance (ANOVA) [37]

We emphasize that features selection depends on the available data. In order to take care of this aspect, we perform *M* replicates selecting each time randomly 70% of the available data. For each algorithm, we choose the features selected more than 80% of the times over the *M* replicates.

**Algorithm 2 Minimal features set construction**

1: Given the target set in a table *m*–by–(*n* + 1) = 466 × 28

2: **for** *k* = 0, 1, …, *M* **do**

3:  Randomly extract the 70% of the target set:

4:  Apply the four different feature selection algorithms;

5: **end for**

6: **for** each algorithm: **do**:

7:   rank the features according to the number of times selected over the *M* runs;

8:   choose the features selected more than 80% of the runs.

9: **end for**

10: Choose the features selected by more than 3 algorithms out of the 4

We ran the feature selection procedure as described in Algorithm 2 and Table 11 for each of the four algorithms we indicate with a “x” the features selected at least in the 80% of random runs. The last column counts how many algorithms selected the feature.

**Table 11:** Features ranking.

Variables	name	L2 SVM	L1 SVM	MI	ANOVA	Final score
*x*_4_	Pelvic torsion_DL-DR	x	x	x	x	4
*x*_5_	Pelvic inclination_(dimple)	x	x	x	x	4
*x*_7_	Inflexion point_ICT /trunk length_VP-DM	x	x	x	x	4
*x*_9_	Inflexion point_ITL /trunk length_VP-DM	x	x	x	x	4
*x*_10_	Lordotic apex_LA_(VPDM) /trunk length_VP-DM	x	x	x	x	4
*x*_13_	Lumbar Fléche_(Stagnara)	x	x	x	x	4
*x*_17_	Surface rotation_(rms)	x	x	x	x	4
*x*_25_	Lateral deviation_VPDM_(+max)	x	x	x	x	4
*x*_11_	Inflexion point_ILS /trunk length_VP-DM		x	x	x	3
*x*_15_	Lordotic angle_ITL-ILS_(max)		x	x	x	3
*x*_16_	Pelvic inclination	x		x	x	3
*x*_19_	Surface rotation_(+max)	x	x		x	3
*x*_20_	Surface rotation_(-max)	x		x	x	3
*x*_26_	Lateral deviation_VPDM_(-max)	x	x	x		3
*x*_1_	Trunk inclination_VP-DM		x		x	2
*x*_2_	Lateral_flexion_VP-DM	x		x		2
*x*_6_	Pelvis rotation	x			x	2
*x*_8_	Kypothic apex_KA_(VPDM) /trunk length_VP-DM	x		x		2
*x*_12_	Cervical Fléche_(Stagnara)			x	x	2
*x*_14_	Kyphosis angle_ICT-ITL			x	x	2
*x*_18_	Surface rotation_(max)	x	x			2
*x*_21_	Surface rotation_(width)	x			x	2
*x*_24_	Lateral deviation_VPDM_(max)			x	x	2
*x*_27_	Lateral deviation_(width)	x	x			2
*x*_3_	Pelvic obliquity_DL-DR			x		1
*x*_23_	Lateral deviation_VPDM_(rms)				x	1
*x*_22_	Pelvic torsion					0

We propose to select as the most significant features those ones that are selected by at least 3 out of the 4 algorithms and we define the *minimal features set* as the set of these features. They are the first 14 features listed in Table 11, namely:

$$\text{Minimal}\;\text{Set}=\{x_{4},x_{5},x_{7},x_{9},x_{10},x_{11},x_{13},x_{15},x_{16},x_{17},x_{19},x_{20},x_{25},x_{26}\}.$$

To check the effectiveness of selection procedure with respect to standard statistic tools, we perform a selection by solely analysing Pearson’s correlation matrix as reported in Table 7 and selecting those features which have a Pearson correlation with the output ≥0.2. Pearson’s selected features are

$$\{x_{1},x_{5},x_{7},x_{8},x_{9},x_{10},x_{11},x_{13},x_{15},x_{16},x_{17},x_{20},x_{21},x_{23},x_{27}\}.$$

If we add also Pearson’s selection in the voting procedure for choosing the most prominent features and we consider those features that obtained at least four votes we obtain the set

$$\{x_{4},x_{5},x_{7},x_{9},x_{10},x_{11},x_{13},x_{15},x_{16},x_{17},x_{20}\}.$$

We name this set *Pearson minimal set*. We note that the Pearson minimal set is not the intersection of the Pearson’s feature set and the minimal features set. Indeed, the feature *x*_4_ (Pelvic torsion_DL-DR) has a low value of the Pearson coefficient (0.11) and it would not be selected by solely the Pearson selection rule, but it is instead selected by all the other four algorithms.

After the features’ selection has been performed, we have available four training sets, namely the full dataset, the minimal set, the Pearson set and the Pearson minimal set, with the same number of samples but with a decreasing number of features.

In the numerical test, we use the four different sets to check the impact of the feature selection over the performance of the learners. we report only the comparison between the full set and the minimal set, because the features selection corresponding to the minimal set led to the highest KPIs.

Results reported in the next sections show that such a reduction in the dimensionality does not strongly affect performance of the ML which is measured by the KPI indicators suggesting that features in Table 6 have a key role in classifying scoliosis.

## Results and discussion

### Recap of the procedure and toolboxes

We recap the overall procedure that led to the final learning model.

1. **Data management**
  - Data acquisition.
  - Data Balancing. Since healthy/scoliotic classes are not balanced we randomly oversampled the less numerous class
  - Data cleaning and normalization.
2. **Features selection**.
  - Features Ranking using different models
  - Features reduction
3. **Training of unsupervised classifiers**.
4. **Training of supervised classifiers**.
  - Hyper parameters tuning of supervised classifiers.
  - Parameters tuning of supervised classifiers.
5. **KPIs analysis and clinical implications**.

For the numerical testing we use standard tools available for unsupervised and supervised training. In particular, the Python’s ScikitLearn [33] library which is an open-source ML library, for clustering and SVM. Specifically, ScikitLearn’s ‘cluster. KMeans’ package with *K* = 2, with the ‘k-means++’ initialization [26] for clustering, and LIBSVM [28] for SVM. For training the DNN we use ADAM [38] as implemented on Keras [39] over TensorFlow paradigm [40].

In all the reported results, we used in Algorithms 1 and 2 *M* = 100 times.

In addition to the standard assessment of the system’s performance we design experiments to check the role of each step of the procedure. In particular:

1. to quantify the effect of features selection we perform the experiments using the target data (after cleaning) with the full set of features and the reduced one;
2. to check the intrinsic information in the data we perform unsupervised clustering both on the full target set and on the reduced one;
3. to check the effect of different value of hyper-parameters tuning we test linear and Gaussian kernel in SVM classification and deepness and wideness in DNN, by choosing different values of *L*, *N*^ℓ^, and the activation function selecting either the ReLu or the sigmoid function. The ReLu performs significantly worst. Hence we report the results only for the sigmoid.

### Performance of unsupervised classifiers

We first apply an unsupervised learning process to the full and minimal target set where the labels are taken out.

We report the results in terms of accuracy and confusion matrix. Accuracy achieved using the full set of 27 features and only the 14 selected features of the minimal set 61.7% and 72.2%, respectively. It is interesting to see how accuracy increased with the features reduction. In fact, this was foreseeable as a smaller size makes the task easier for a learning machine that does not exploit labels to group samples into clusters.

In both the experiments, false positives and false negatives are well balanced, showing the robustness of the target data, namely that the rastereographic information allow to clearly separate the two clusters, i.e. subjects with scoliosis and healthy ones.

### Performance of supervised classifiers

**Fig 3:**
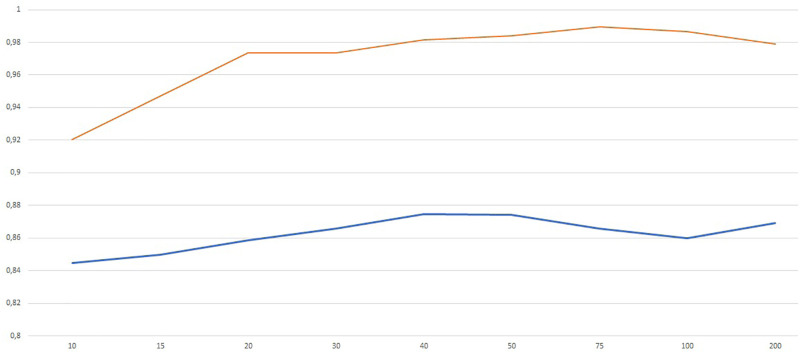
Validation (blue) and training (red) accuracy for increasing number of neurons in the shallow network (one hidden layer).

We first trained the DN with the sigmoid activation function on the full data set using different architectures with the aim of exploring the role of wideness and deepness. In particular, we trained a shallow network (i.e. one hidden layer) with increasing number of neurons *N* in order to understand the role of wideness. We increased the number of neurons *N* from 5 to 200 and we report the results in terms of average validation accuracy (blue) and training accuracy (red) in Fig 3. Average classification accuracy is almost everywhere higher than 80%, being 40 ≤ *N* ≤ 50 the best range of neurons. We observe that the training accuracy reaches almost value 1 for *N* ≥ 50. In Table 12 we report the accuracy and balanced accuracy of the best configuration which corresponds to *N* = 40.

**Table 12:** Accuracy (ACC) and Balanced Accuracy (BACC) obtained by two DNs used in the experiments using either the full set or the minimal set of features. *L* − 1 is the number of hidden layers and *N*^*ℓ*^ are the neurons per layer.

	(*L* − 1, *N*^*ℓ*^)	ACC	BACC
**Full set**	(1,40)	87.5%	87.4%
	(2,20)	86.3%	86.6%
**Minimal set**	(1,40)	83.7%	83.4%
	(2,20)	85.5%	85.5%

**Fig 4:**
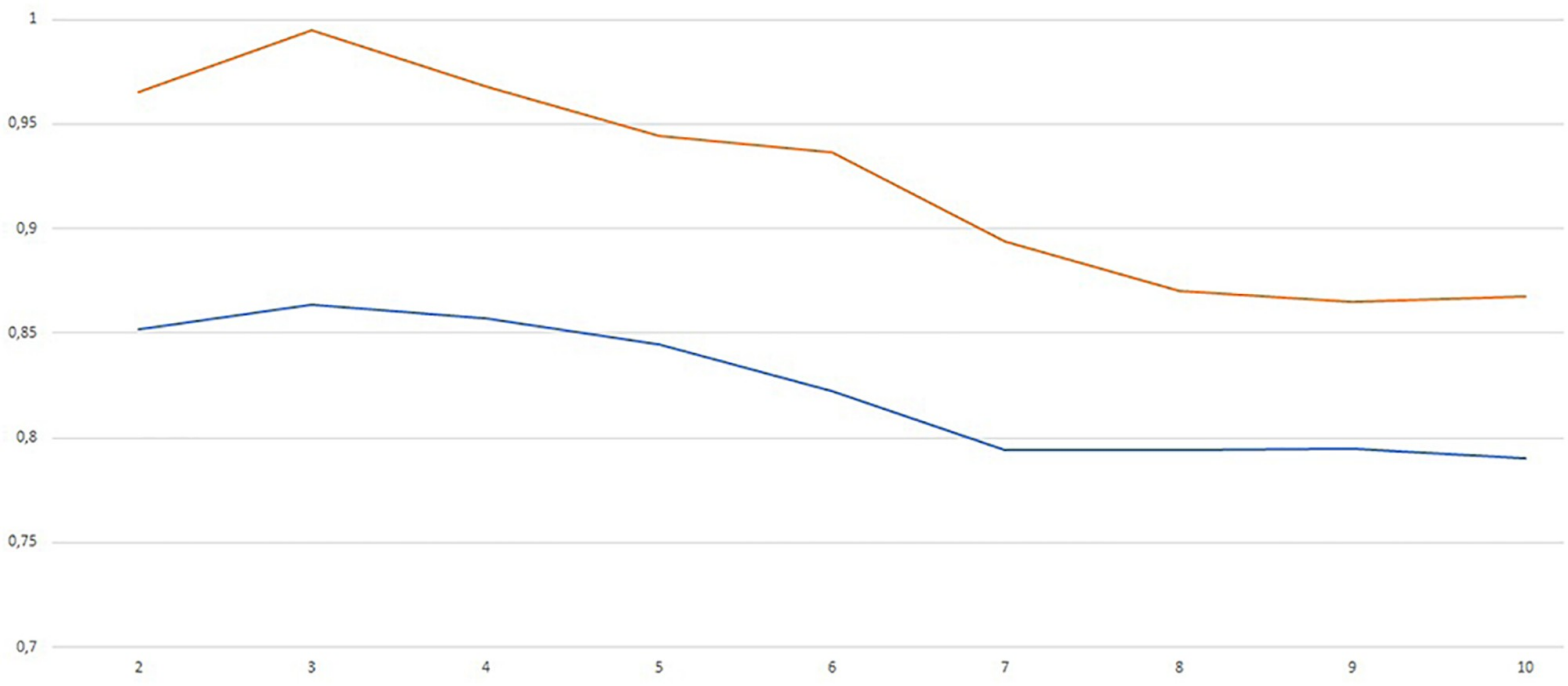
Validation (blue) and training (red) accuracy for increasing number of layers with *N*^*ℓ*^ = 20 for all *ℓ* = 1, …, *L*.

We also performed a test increasing the number of layers from *L* = 2 to *L* = 10 with a fixed number of neurons per layer *N*^*ℓ*^ = 20 for all *ℓ* = 1, …, *L*. The results are in the Fig 4.

The best results correspond to *L* = 3 (two hidden layers). The picture shows that increasing network deepness do not produce better results. This may be due also to the well known difficulties in training such deep networks using gradient based method like Adam. The accuracy and balanced accuracy are reported in Table 12.

We also analyze the performance when using the minimal features set with only the 14 features described in *Features selection procedure* section.

From the results it emerges that the performance using the minimal set decreases only by 2–3%.

We also used the nonlinear SVM classifier as implemented in LIBSVM with the kernel chosen as RBF function with spread *γ*. The best values of the hyper-parameters *C* and *γ* have been set with the tuning procedure. The procedure has been replicated both for the full set and for the minimal set of features. Resulting values of *C* and *γ* are reported in Table 13.

**Table 13:** Parameters of SVM defined by the tuning procedure.

	Full	Minimal
*C*	10	10
*γ*	10^−3^	10^−2^

Classification accuracy and balanced accuracy reached by the SVM classifier both for the full and the minimal sets of features are reported in Table 14.

**Table 14:** Accuracy (ACC) and Balanced Accuracy (BACC) of SVM.

	ACC	BACC
**Full set**	84.9%	84.7%
**Minimal set**	82.2%	81.5%

Accuracy is higher in supervised learning than the unsupervised one, as expected since the learner can exploit more information (i.e. the labels). Both DNs and SVMs have accuracy and balanced accuracy over 80%. However DNs are almost over 85%, being preferable as classifiers between AIS and healthy patients.

We observed that a reduction of the input dimensionality by almost 50% brought only a slight deterioration of the accuracy and balanced accuracy when using either DNs or SVMs. This seems to suggest that the 14 features selected in the minimal set bring the most significant information. Indeed the physicians analyzed them and the clinical comments are reported at the end of the paper.

### Clinical comments and limits

We analyze in this section the 14 features (i.e. rasterstereography parameters) identified by means of the feature selection process with the aim of verifying their clinical role. Among the identified parameters reported as the first 14 in Table 11 there are measures on the three planes lateral, sagittal and frontal. Five of the selected parameters, identified by the *x*_17_, *x*_19_, *x*_20_, *x*_25_, *x*_26_, are commonly related to the evaluation and diagnosis of scoliosis, in fact lateral deviation and vertebral rotation are well known clinical signs of the disease. Indeed scoliosis is defined as a lateral curvature of more than 10 degrees as measured by the Cobb Technique on standing anterior posterior radiography of the spine. Scoliosis is a complex three-dimensional spinal deformity, that forms a complex curve leading to deformities not only in the coronal plane but in all three planes, which is caused by the self-rotating movement of the spine. An important feature of idiopathic scoliosis deformity is the vertebral axial rotation which accompanies the vertebral lateral deviation. Mechanical interactions within the spine have been implicated in causing vertebral rotation with lateral deviation. This rotation is thought to be significant for initiation and progression of scoliosis. The magnitude of vertebral axial rotation correlates with lateral deviation of vertebrae from the spinal axis, and the rotation is maximal near the curve apex ([41–44]).

Nine parameters, identified by the *x*_4_, *x*_5_, *x*_7_, *x*_9_, *x*_10_, *x*_11_, *x*_13_, *x*_15_, *x*_16_, are instead related to the sagittal plane and these results may seem unexpected because the scoliosis is predominantly characterized by alterations on frontal and trasversal planes. However recent clinical papers seem to suggest a role of these parameters. Sullivan et al. [45] underlined the importance of sagittal plane and the need of a global assessment in the evaluation of scoliosis. They find a strong correlation between scoliosis severity and loss of 3D kyphosis. Increasing severity of coronal plane curvature is associated with a progressive loss of thoracic kyphosis. Moreover, previous studies have suggested that thoracic hypo-kyphosis is a primary event in the development of Idiopathic Scoliosis (IS) [46]. Nevertheless, because of historic restrictions of planar imaging of this multidimensional deformity, there is little information regarding the correlation of thoracic kyphosis with the increasing severity of idiopathic scoliosis. Modern, low-radiation-exposure 3D imaging systems have now made routine clinical 3D imaging feasible. These imaging modalities offer the possibility to study the components of the scoliotic deformity in the planes of origin for each vertebra, free of the distortions on 2D images [7]. Sullivan et al. [45] found a strong linear correlation between the magnitude of the main thoracic coronal curve and loss of 3D thoracic kyphosis. Three of these sagittal parameters, *x*_4_, *x*_5_, *x*_16_, are related to sagittal alignment of the pelvis, but this was expected since an influence of the sagittal parameters of the column in the identification of patients is known. Legayeet et al. [47] demonstrated the key importance of the anatomical parameter of pelvic incidence in the regulation of the sagittal curves and this is maintained when the scoliosis disease occurs. Moreover, Fei Han et al. [48] underlined that patients with degenerative scoliosis (DS) may have a higher pelvic incidence, which may impact the pathogenesis of DS. In fact an unbalancing of pelvic incidence should cause scoliosis if the degeneration speed of the two sides differs [49]. The limits of traditional 2D imaging have restricted the evaluation of the adolescent idiopathic scoliosis effects on the sagittal plane, in fact it is impossible to perform simultaneous evaluation of the frontal and sagittal profiles of the spine. In addition, when sagittal evaluation is performed, the patient is asked to put arms forward, which is a non-natural position. Since RX evaluation exposes the patients to ionizing radiations, it can not be made frequently in clinical follow up frequency.

From our results we could assume that, in order to preform a valid screening and to easily permit the scoliosis diagnosis, ML could be a valid method. In fact, a rapid and semi-automatic ML-based screening may help the clinician to detect scoliosis earlier and rapidly. With an early diagnosis it is possible to treat the patients promptly in order to contain the development of the scoliotic curve. In every screening campaign, especially in young people, it is important not to use invasive methods such as X-rays. The idea from which our study started was precisely to apply ML techniques to optimize the use of a non-invasive method to be used in the screening of patients suffering from scoliosis. It should also be emphasized that one of the main drawbacks of using the Rx parameter (Cobb angle) for assessing and monitoring scoliosis is that it does not measure the deformity in a three-dimensional (3-D) space. Therefore, it is of interest to develop reliable and noninvasive methods to monitor the three-dimensional (3-D) progression of scoliosis [15].

The rasterstereography provides a three-dimensional reconstruction of the spine curvature and the patient can assume a natural position. Moreover it provides a dynamic evaluation: indeed, in six seconds, twelve frames are recorded and the average results are obtained. So it is particularly suited to the sagittal plane parameters of the spine without any ionizing radiations exposure and any risk for the patient [50]. Summarizing, the features selected by the procedure seems to have some clinical usefulness thus validating the overall learning procedure based on DNN or SVM.

It should be emphasized that purely clinical features, such as e.g. Adam’s bending test, rib cage abnormalities, rib hump etc. were not considered in our analysis. This might constitute a limitation of our study, although the main aim of our study was to evaluate the possible use of the solely Formetric^™^ data in scoliosis screening. This clinical data can be integrated in a successive steps by combining different databases.

## Conclusion

In this study it has been showed that both supervised and unsupervised learning using rasterstereography data give high accuracy results in classifying AIS patient versus healthy one. As expected the accuracy is higher in the supervised case. However the use of clustering procedure allowed to group patients in well separated clusters which showed strong intra-group similarity. The supervised algorithms used, both Deep Networks and SVMs, performed quite well with accuracy over 80%.

Those evidences confirm as data mining can represent a new approach to identify patients with AIS from healthy ones Moreover clustering procedure can represent a useful method to classify AIS patient after rasterstereography collecting the maximum available data for the patient relating them each other in a set of category. Finally our results confirm that a subset of rasterstereography parameters can be used in the screening of AIS patients, although X-ray imaging cannot be replaced by this method.

Rasterstereography tool can be used to perform a scoliosis screening in order to improve the selection of patient that need to underwent X-ray examination. Furthermore thanks to the fact that Rastereography machine is easily carried, it can be used to propose once again scholar screening for pre-adolescent pupils.

## Supporting information

S1 Data (XLSX)
